# Tacrolimus Associated Posterior Reversible Encephalopathy Syndrome – A Case Series and Review

**DOI:** 10.4084/MJHID.2014.014

**Published:** 2014-02-18

**Authors:** Susmitha Apuri, Kristin Carlin, Edward Bass, Phuong Thuy Nguyen, John N. Greene

**Affiliations:** 1 Internal and Hospital Medicine, Moffitt Cancer Center, University of South Florida College of Medicine, 12902 Magnolia Drive, Tampa, Florida 33612-9497, USA; 2 Department of Neurology, Moffitt Cancer Center, University of South Florida College of Medicine, 12902 Magnolia Drive, Tampa, Florida 33612-9497, USA; 3 Department of Infectious Diseases. University of South Florida College of Medicine, 12902 Magnolia Drive, Tampa, Florida 33612-9497, USA; 4 Infectious Diseases and Hospital Epidemiologist, Moffitt Cancer Center, University of South Florida College of Medicine, 12902 Magnolia Drive, FOB-3, Tampa, Florida 33612-9497, USA

## Abstract

Tacrolimus is an immunosuppressive drug mainly used to lower the risk of transplant rejection in individuals who are post solid organ or hematopoietic transplantation. It is a macrolide which reduces peptidyl-propyl isomerase activity and inhibits calcineurin, thus inhibiting T-lymphocyte signal transduction and interleukin-2 (IL-2) transcription. It has been associated with Posterior Reversible Encephalopathy Syndrome (PRES), a disease of sudden onset that can present as a host of different symptoms, depending on the affected area of the brain. While infectious causes of encephalopathy must always be entertained, the differential diagnosis should also include PRES in the appropriate context. We report three cases of PRES in patients with acute myeloid leukemia (AML) placed on tacrolimus after receiving a bone marrow transplant (BMT). The focus of this review is to enhance clinical recognition of PRES as it is related to an adverse effect of Tacrolimus in the setting of hematopoietic transplantation.

## Introduction

Tacrolimus is a macrolide lactone derived from the bacteria Streptomyces tsukubaensis, which is used in the treatment of various immune-mediated disorders. This drug binds with cyclosporine to form a drug-receptor complex. This complex then competitively binds to and inhibits calcineurin. It functions by inhibiting the transcription of Interleukin-2 (IL-2) by T-helper lymphocytes, as well as inhibiting T-helper lymphocyte growth and proliferation.^17^–^18^ Unfortunately, tacrolimus is associated with renal and neural toxicity, among its other side effects of immunosuppression. One of the more uncommon presentations of neurotoxicity is posterior reversible encephalopathy syndrome (PRES). PRES was initially described by Hinchey et al^1^ in 1996 as a clinical neuroradiological entity. It is characterized by generalized seizures, headache, vision changes, coma, paresis, hemianopsia, nausea, altered mental status, and/or focal neurological deficits.^2^ There are varying reports of neurotoxicity related to tacrolimus ranging from 7 to 32% in solid organ transplants (SOT).^47^ The overall incidence of PRES occurs in 0.5%–5% of SOT recipients and is most commonly associated with tacrolimus.^50^ We are describing 3 cases of neurotoxicity related to tacrolimus in the hematopoietic transplant population.

## Patient One

Our patient is a 36-year-old white female with a history of AML, who underwent allogeneic BMT 41 days prior to the onset of her neurologic symptoms. Her posttransplant course was complicated by mild graft versus host disease (GVHD) of the gastrointestinal tract and skin, for which she was placed on tacrolimus on day 16. On day 29, she was also started on prophylactic trimethoprim-sulfamethoxazole (TMP-SMX) for pneumocystis jiroveci pneumonia (PCP). Additionally, she was diagnosed with cytomegalovirus (CMV) reactivation and BK virus hemorrhagic cystitis. She was placed on valgancyclovir (day 19) and Cidofovir (day 27) respectively. She subsequently developed acute renal failure with creatinine levels peaking at 3.7 mg/dL and BUN peaking at 88 mg/dL on day 34. Cidofovir was presumed to be the likely causative agent for the acute kidney injury; although she had recently been placed on multiple additional nephrotoxic agents, including the aforementioned tacrolimus, TMP-SMX, and pyridium.

**Figure 1:**
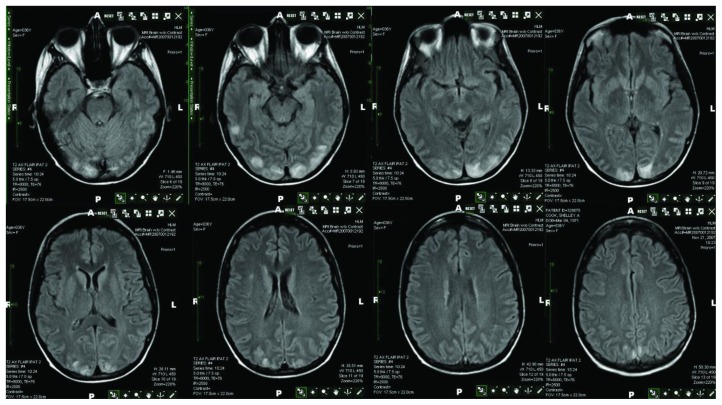


On day 41, she presented with sudden onset of altered mental status manifesting as staring into space and right gaze preference. Tonic-clonic activity, incontinence, or tongue biting was not observed, but concern was raised for new onset of seizures given the episode of unresponsiveness with fixed gaze. She was started on treatment with 2 mg of intravenous lorazepam (AtivanTM) and 500 mg of levetiracetam (KeppraTM). She underwent imaging of the brain with a non-contrast computed tomography (CT). The CT scan did not demonstrate any acute findings. A non-contrast magnetic resonance imaging (MRI) was then obtained. The MRI study showed increased FLAIR and T2 signal in the cortex of the infero-posterior, posterior temporal and occipital lobe with absence of diffusion abnormality consistent with PRES (Figure 1). An EEG was negative for epileptiform activity. Cerebrospinal fluid analysis was performed for possible encephalitis as well as lepto-meningeal recurrence with negative results.

Interestingly, the patient was taken off of tacrolimus on day 34 at the onset of acute renal failure, prior to the onset of PRES. The tacrolimus levels were within the desired range of 10–15 ng/ml throughout this course.

She was switched to sirolimus one week after the onset of PRES, maintaining serum levels within the desired range of 8–12 ng/ml. At this time her neurological symptoms had improved although not completely resolved. Three weeks following the diagnosis of PRES, a repeat MRI with gadolinium was performed showing resolution of the posterior areas and a small focus on FLAIR in the right frontal lobe. Levetiracetam was discontinued and sirolimus was maintained. The patient was followed for an additional year. She had a complete recovery from this event. No additional seizures or new neurological findings occurred during this time.

## Patient Two

Altered mental status and severe headache developed in a 54 year old female, previously diagnosed with acute myeloid leukemia, four weeks after admission for high dose chemotherapy followed by matched unrelated donor stem cell transplant. Following her conditioning chemotherapy, which consisted of busulfan and fludarabine and subsequent bone marrow transplant, she developed a nodular pneumonia thought to be fungal in nature but culture negative by bronchoscopy specimens. During this time, the patient was on tacrolimus for graft versus host disease prophylaxis. This was started 25 days prior to the onset of mental status changes. Tacrolimus levels were maintained in a therapeutic target range of 10–15ng/ml during this time frame. On day 27 of the admission, she complained of severe headache which was followed by confusion. Also of note, the patient developed accelerated hypertension in the 48 hours surrounding the episode of confusion. Neither visual disturbance nor seizure activity was noted. Prior to this event, the patient was fairly normotensive with a systolic blood pressure range of 120–130 mmHg. At the peak of confusion, she was noted to have blood pressure of 188/90 mmHg. Calcium channel blocker therapy was then started and normotension was achieved.

Cranial T2-weighted MRI showed increased FLAIR and signal in the left and right cerebellar hemispheres that did not enhance or show restricted diffusion. There was also increased flair signal in the left and right posterior parietal occipital cortex consistent with posterior reversible leukoencephalopathy. Tacrolimus was discontinued and mycophenaolate mofetil was initiated. Within 24 hours of discontinuation of the tacrolimus, the neurologic symptoms began to resolve and the patient was again normotensive. Subsequent to this, she did develop grade II graft versus host disease of skin requiring high dose steroids and sirolimus was then added to the regimen. Because of the concern for potential worsening of the nodular pneumonia, steroid taper was initiated as soon as clinically feasible. Ultimately, she recovered and was discharged from the hospital in stable condition, with resolution of all neurologic findings.

## Patient Three

Our third patient with a history of AML, refractory to chemotherapy, underwent a matched unrelated donor BMT. On transplant day 2, the patient was started on tacrolimus for graft versus host prophylaxis. Tacrolimus levels were maintained between 15.2 ng/ml and 17.3 ng/ml. Concurrently, she was found to have confusion, visual hallucinations, headaches, gait disturbances, nausea and vomiting. CT scan of the head showed no acute findings. These complaints were soon attributed to hyponatremia of 121mEq/L. As the sodium was gently corrected, her mental status returned to normal baseline within three days.

On transplant day 6, the patient again developed neurologic changes including visual disturbances, cerebellar ataxia, and headache. The visual disturbance consisted of seeing flashing lights as well as the inability to distinguish faces or colors. She had labile blood pressure throughout the hospital stay with systolic blood pressures ranging from 120mmHg to 180mmHg. CT scan of the head showed decreased attenuation in the bilateral mesial aspect of posterior parietal cortex as well as occipital cortex. MRI showed new areas of increased signal on the cortical gyral surfaces of both occipital lobes and portions of the temporal lobes. There was no diffusion restriction. There was no evidence of hemorrhage or acute stroke. These findings were consistent with PRES. EEG showed moderately severe slowing of the background rhythm, consistent with encephalopathy. There was no evidence of focal abnormalities or epileptiform discharges.

Given the diagnosis of PRES, the tacrolimus was discontinued cyclosporine and mycophenalate mofetil were initiated. By the time of her discharge on transplant day 20, her mental status improved significantly however she was left with a broad based gate. This was thought to be due to busulfan toxitcity. Mycophenalate mofetil was discontinued once engrafted and rapamune initiated. Cyclosporine was continued upon discharge. On day 46, she was readmitted to the hospital with alteration in mental status, hyponatremia and was found to have GVHD grade 1 of the duodenum and Grade 1–2 GVHD of the rectum. Infectious work up was negative as a cause of encephalopathy. During this admission, the patient remained normotensive. Repeat MRI showed evidence of recurrent PRES and was thought to be now related to cyclosporine. Unfortunately, the patient continued to decline and enrolled in hospice services, dying shortly thereafter.

## Discussion

AML is a disease of the bone marrow where abnormal cancer cells grow rapidly and replace healthy marrow. This process increases the susceptibility for bone pain, bleeding and infections, anemia and recurrent fevers. Initial treatment almost always consists of chemotherapy regimen, such as duanorubicin and cytarabine. BMT is an option often reserved for chemotherapy failure. Three distinct modalities of BMT include autologous transplant, allogeneic transplant and umbilical cord transplant. In allogeneic transplant, hematopoietic stem cells are collected from a donor and ultimately infused into a patient after high-dose chemotherapy. Complications from this procedure include immunosuppression, anemia, bleeding, fever, and infections as well as rejection or GVHD.^19^

The primary role of tacrolimus (also FK-506, or Fujimycin) is to prevent GVHD in the post-allogeneic solid organ transplant patient. In recent years, it has been increasingly used as an immunosuppressive agent in hematopoietic stem cell transplantation as well. Neurotoxicity secondary to tacrolimus has been well described, particularly in solid organ transplant recipients.^15^

PRES, as the name suggests, is a constellation of symptoms associated with vasogenic edema, most commonly, of the posterior cerebral vasculature, often affecting the parietaloccipital region. Other vascular territories can also be affected in PRES, such as the posterior portion of frontal lobe and temporal lobe.^23^ The abnormalities primarily affect white and gray matter, but the cortex can also be involved. Although the diagnosis may be suggested by CT, MRI of the brain is the most sensitive diagnostic tool.^3^ Distinguishing between vasogenic edema in PRES and cytotoxic edema in the setting of cerebral ischemia can be reliably determined by diffusion weighted MR imaging.^46^ PRES is typically a reversible phenomenon, as indicated by the name, but if not recognized early and treated appropriately, permanent brain injury may ensue.^8^ Many agents and etiologies have been linked to PRES (Table 1).

PRES is an increasingly recognized neurologic disorder with characteristic image findings. Clinically, its presentation can be variable and the differential is wide (Table 2). The pathophysiology of PRES still remains unclear. The mechanism of action of Tacrolimus induced PRES may be similar to Cyclosporine. Neurotoxicity associated with Cyclosporine was thought to be facilitated by hypomagnesemia, hypocholesterolemia, hypertension and the vasoactive agent endothelin.^53^ Cyclosporine is also believed to exacerbate hypertension by inhibiting nitric oxide production.^54^ The mechanism of cellular injury is thought to be secondary to mitochondrial dysfunction as the symptoms of Cyclosporine induced neurotoxicity and mitochondrial encephalopathy appear to be similar.^55^ Schwartz et al assessed sixteen patients by evaluating the factors responsible for the neurotoxic effects of Cyclosporine using neuroimaging as well as clinical and laboratory data. The only major factor associated with neurotoxic effects of Cyclosporine was systemic hypertension although thrombocytopenia, microangiopathic hemolytic anemia and hypoalbuminemia are common.^56^ The syndrome is likely initiated by a breakdown in the blood brain barrier leading to a leakage of fluid into the interstitium of the brain tissue and the development of vasogenic edema.^9^ In addition, immunosuppressant drugs exert cytotoxic effects on the vascular endothelium.^10^,^11^ It is unknown whether vasospasm plays a clear role in the genesis of local ischemia.^12^ Sympathetic innervation regulates the vessels of the brain during acute elevations in blood pressure. Because auto-regulatory mechanisms are dependent on the neurogenic response, the more poorly innervated areas in the posterior circulation are more vulnerable to increased blood pressure.^12^,^13^ As PRES may occur in patients with hypertensive encephalopathy where the limits of autoregulation are exceeded, hypertension has been suggested as a potential risk factor for neurotoxicity induced by tacrolimus and other drug related cases of PRES. It is important to exclude unrecognized increases in blood pressure that can be overlooked or occur in the outpatient setting. Two of our three patients were hypertensive surrounding the PRES event and a careful review of the chart records failed to show any elevations in the third case.

Unfortunately, immune suppressant blood levels do not appear to correlate with severe neurotoxicity or PRES, but discontinuation or change in the offending immunosuppressant can lead to clinical improvement. Grimbert et al reported significant levels of Tacrolimus in the CSF which suggests that this molecule can cross the blood brain barrier. Demyelination was noted in two cerebral biopsies associated with leukoencephalopathy in patients who underwent liver and lung transplantation.^57^

Immune challenges post-transplant (i.e. transplant rejection, GVHD), effects of chemotherapy, and the risks of infection in the immunosuppressed state may further contribute to toxicity. Clearly, a balance exists between adequate immunosuppression and infection risk.^14^ The diagnosis of tacrolimus-associated PRES was made by the following criteria: (1) characteristic clinical findings (headache, mental status changes, seizures, visual abnormalities and/or focal neurological deficits) with the exclusion of other possible causes (i.e. infection, metabolic disturbances and structural neurological lesions) and (2) characteristic findings of subcortical white matter lesions on CT or MRI of the brain.^16^

Our patients presented with classic MRI findings of PRES at the onset of symptoms, and had appropriate response to treatment in the expected timeframe. Patients affected by PRES may experience some or all of the following: visual disturbances, altered mental status, seizures, and headaches. Of note, the association of hypertension with PRES is well reported; however is not always a component of PRES in patients on immunosuppressive therapy.^49^

These cases demonstrate the challenge of understanding the pathophysiology of PRES, since tacrolimus use would appear to be the main contributor. However, in the first case, PRES did not appear until four days after this medication was withheld secondary to acute renal failure. Although subtherapeutic levels of immunosuppressants have been reported as causing PRES, observations of the onset of this disease at the time of declining levels is seemingly paradoxical. There exists the possibility of delayed onset of symptoms, or perhaps the confounding factor was related to the acute renal failure itself. Proteinuria and hypoalbuminemia are known to be associated contributors to the development of PRES. The time course of developing PRES from the onset of beginning immunosuppressant therapy is particularly variable; therefore, the development of PRES, even while maintaining therapeutic levels, is not surprising.

In addition, our third patient had recurrent PRES with the second event likely related to cyclosporine. Interim images had revealed resolution of PRES once the offending Tacrolimus had been discontinued. Cyclosporine was subsequently initiated and maintained in the therapeutic target range. Cyclosporine has been reported in the literature to be an inciting factor in PRES events.^51^ There have been published case reports documenting recurrent episodes of PRES with varying inciting factors.^52^

## Conclusions

Tacrolimus-associated PRES is an uncommon, but serious complication after BMT, especially with matched-unrelated or mismatched transplants, where higher levels of immunosuppression are required to prevent rejection. It should also be noted that supratherapeutic levels of tacrolimus need not be present to cause a PRES event.^48^

This syndrome should be promptly recognized as it is potentially reversible and generally responds to withholding or decreasing the dose of tacrolimus in addition to controlling hypertension and seizures.

Although there are common yet heterogeneous etiologies that surround the onset of PRES, more research into the mechanisms surrounding PRES is needed to understand this disease process. The compilation of these diseases and a study of the time course of the patient’s history leading up to and during the presentation of PRES may better elucidate the means by which we treat and diagnose this disease.

## Figures and Tables

**Table 1:** Common causes of PRES

Medications	Chemotherapy agents	Cytokines and Immunoglobulin’s	Others

Linezolid^20^	Cyclosporine^4^,^5^	Rituximab^37^	Blood transfusion^44^
Antiretroviral^21^	Cisplatin^29^	Infliximab^38^	IV contrast agents^23^
Erythropoietin^22^	Oxaliplatin^30^	Interferon-alpha^39^	Hypertension^23^
Cocaine^23^	Carboplatin^31^	Interleukin-2^40^	Pre-eclampsia^23^
Ephedra^24^	Gemcitabine^29^	Etanarcept^41^	Eclampsia^23^
Lysergic acid	Cytarabine^32^	Anti-lymphocyte	Tumor lysis syndrome^8^
Amide^25^	Methotrexate^33^	globulin^42^	Sepsis^8^
Carbamazepine^26^	Vincristine^34^	Tacrolimus^1^,^23^	SLE*^23^
Intravenous	Irinotecan	Sirolimus^43^	TTP*^23^
Caffeine^27^	Hydrochloride^35^		ITP*^23^
Corticosteroids^23^,^28^	Bevazicumab^35^		Anti-depressants^8^
	Sunitinib^36^		Renal Failure^8^

* SLE = Systemic lupus erthythematosus; TTP = Thrombotic thrombocytopenic purpura; ITP = Idiopathic thrombocytopenic purpura.

**Table 2:** Differential Diagnosis of PRES:

***Emergent****- Ischemia, Thrombosis, Mass effect, Hemorrhage, Hydrocephalus, Seizures, Trauma.*
*I****nfectious***- Encephalitis, HIV*,PML*,SSPE*, Toxoplasmosis, Neurosyphilis, Septic Cerebral Embolism,Rubella, Lyme Disease.
***Inflammatory***- SLE*, Scleroderma, Vasculitis, Multiple sclerosis.
***Other****- Toxic white matter demyelination, Adrenoleukodystrophy, Uremic encephalopathy.*

* HIV-Human Immunodeficiency Virus; PML=Progressive Multifocal Leukoencephalopathy; SSPE=Subacute Sclerosing Panencephalitis; SLE=Systemic Lupus erythematosus^45^
